# Lymphocyte-To-Monocyte Ratio is Partially Mediated in Age-Related Cardiovascular Mortality in HFpEF: Immunosenescence, Inflamm-Aging, and Longevity

**DOI:** 10.31083/RCM45403

**Published:** 2026-02-11

**Authors:** Xiaojie Cai, Menghui Liu, Chong Feng, Sanhua Tang, Peng Qin, Yubin Li, Teng Wang, Lixiang He, Jiangjie Lei, Yi Zhou, Yue Guo, Xiaodong Zhuang, Xinxue Liao

**Affiliations:** ^1^Department of Cardiology, The First Affiliated Hospital, Sun Yat-sen University, 510080 Guangzhou, Guangdong, China; ^2^NHC Key Laboratory of Assisted Circulation and Vascular Diseases, Sun Yat-sen University, 510080 Guangzhou, Guangdong, China; ^3^Department of Cardiology, Liuzhou Municipal Liutie Central Hospital, 545000 Liuzhou, Guangxi, China; ^4^Department of Cardiology, People's Hospital of Wuzhou, 543000 Wuzhou, Guangxi, China; ^5^Department of Cardiology, Deqing People's Hospital, 526000 Zhaoqing, Guangdong, China; ^6^Department of Cardiology, The Second People's Hospital of Changzhi, 046000 Changzhi, Shanxi, China

**Keywords:** lymphocyte-to-monocyte ratio, age, heart failure with preserved ejection fraction, immunosenescence, inflamm-aging

## Abstract

**Background:**

Heart failure with preserved ejection fraction (HFpEF) is recognized as an aging-related clinical syndrome with high mortality, from which systemic inflammation could represent a primary culprit. Thus, this study aimed to evaluate the association between the lymphocyte-to-monocyte ratio (LMR), a systemic inflammation marker, and clinical outcomes, and to explore the mediation effect of the LMR in the relationship between age and mortality for HFpEF.

**Methods:**

Participants in the Real-world Data of Cardiometabolic ProtEcTion trial (RED-CARPET) trial were categorized into tertiles based on the recorded LMRs. We employed Cox regression analyses to explore the relationship between the LMR and mortality, as well as mediation analyses to determine whether the LMR serves as a mediator between aging and mortality.

**Results:**

A total of 1274 inpatients with HFpEF were enrolled between May 2015 and December 2023. After a median follow-up period of 4.9 years, there were 166 recorded deaths, of which 82 were due to cardiovascular causes. In the third model, each one-unit increase in standard deviation (SD) for age was correlated with a 1.98-fold increase in the risk of overall mortality (95% confidence interval (CI), 1.66–2.35) and a 1.73-fold increase in the risk of death due to cardiovascular disease (95% CI, 1.36–2.21). Compared to patients in the first tertile of the LMR, those in the third tertile exhibited a lower risk of death (hazard ratio (HR) 0.42; 95% CI (0.27–0.65)) and cardiovascular death (HR 0.23; 95% CI (0.12–0.46)). Mediation analyses indicated that the LMR partially mediated the relationship between age and cardiovascular mortality in patients with HFpEF, with a mediation proportion of 17.9% (95% CI (7.2%–36%);* p* < 0.001).

**Conclusions:**

The LMR may serve as a marker for mortality and is implicated in the mediation of age-related cardiovascular death in patients with HFpEF. This study offers a cost-effective predictor for HFpEF and suggests potential mechanisms related to immunosenescence and inflammation-related aging (inflamm-aging).

## 1. Introduction

One out of 10 people older than 40 develops heart failure with preserved ejection fraction (HFpEF) worldwide, which results in substantial morbidity and mortality [1, 2, 3]. Studies from large cohorts suggested that patients with HFpEF had a 5-year mortality of 75.7%. Meanwhile, patients aged ≥85 years had a 6-fold higher risk of death compared with patients aged ≤55 years [2, 4]. Immune system decline with age is designated as immunosenescence, characterized by a decreased capacity to surveil and clear senescent cells, alongside elevated levels of cytokines. This phenomenon is commonly referred to as inflammation-related aging (inflamm-aging) [5, 6, 7, 8, 9]. Low-grade systemic and local inflammation, impaired lymphocytopoiesis, and proinflammatory monocyte recruitment have been identified as important links [7, 10, 11, 12, 13]. Inflammation might enhance the risk of major adverse cardiovascular events in a hyperlipidemia-independent way [14, 15]. HFpEF is distinguished by a persistent inflammatory state that involves lymphocytes and monocytes/macrophages [16, 17, 18, 19, 20]. The lymphocyte-to-monocyte ratio (LMR) is considered a systemic inflammatory marker; meanwhile, studies on the prognostic value of the LMR, derived from the blood routine, for mortality in patients with HFpEF are limited. Furthermore, to our knowledge, studies have yet to investigate whether the LMR has a mediating effect in relationships between age and unfavorable outcomes among patients with HFpEF.

Thus, this study primarily aimed to provide clinical evidence that systemic inflammation constitutes one of the important mechanisms linking aging to poor prognosis from HFpEF. Moreover, this study aimed to investigate the role of systemic inflammation in this relationship, to understand the aging mechanism, and identify a target for clinical interventions. Therefore, we utilized data from the RED-CARPET trial (Real-world Data of Cardiometabolic ProtEcTion trial, ChiCTR2000039901) to evaluate the relationship between the LMR and mortality. Additionally, we aimed to determine whether the LMR serves as a mediating factor between age and adverse clinical outcomes in patients with HFpEF.

## 2. Materials and Methods

### 2.1 Study Design and Participants

This research utilized data from the RED-CARPET trial, a real-world study conducted by the Department of Cardiology at the First Affiliated Hospital of Sun Yat-sen University. The trial aimed to assess the relationships between risk factors for cardiometabolic diseases and clinical outcomes and has been registered with the Chinese Clinical Trials Registry (ChiCTR2000039901). This study enrolled 2448 inpatients with heart failure (HF) who had at least one ultrasound cardiogram (UCG) measurement and documented clinical outcomes. The data were collected between May 2015 and December 2023 at the Department of Cardiology, the First Affiliated Hospital of Sun Yat-sen University.

Inclusion criteria included: (1) aged over 18 years; (2) diagnosis of heart failure; (3) at least one UCG measurement; (4) availability of clinical outcomes. Exclusion criteria included: (1) missing left ventricular ejection fraction (LVEF) or LVEF less than 45% (n = 631); (2) absence of baseline covariates (n = 498), including missing smoking history (n = 48), incomplete blood routine data (n = 384), and missing N terminal pro-B-type natriuretic peptide (NT-proBNP)/BNP data (n = 66); (3) presence of severe valvular disease (n = 45).

A total of 1274 eligible patients diagnosed with HFpEF were included (refer to **Supplementary Fig. 1**). This research was conducted based on the Declaration of Helsinki and approved by the Ethics Review Board of the First Affiliated Hospital of Sun Yat-sen University. Clinical data were collected through electronic medical records, while mortality information was obtained from the official death registration system and via follow-up telephone calls.

### 2.2 Data Definitions and Collections

Clinical records included data on age, gender, and self-reported medical history of cardiovascular disease (CVD), atrial fibrillation (AF), hypertension, diabetes, and smoking status, which were subsequently verified by medical staff through examination of medical records, imaging studies, and laboratory tests.

Blood pressure measurements (systolic and diastolic) were recorded in a seated position, with an average of three readings. Anthropometric measurements were conducted by medical personnel, with height measured using a tape measure and weight measured by calibrated scales. Body mass index (BMI) was computed by dividing weight (in kg) by height squared (in m^2^). Smoking status was grouped as never smoked or former/current smoker. Blood samples were collected after an overnight fast of 8 to 12 hours. Laboratory analyses for parameters such as Glycated Hemoglobin A1c (HbA1c), BNP/NT-proBNP, serum creatinine, low-density lipoprotein cholesterol (LDL-c), total cholesterol (TC), high-density lipoprotein cholesterol (HDL-c), and triglyceride (TG) levels were performed using standard techniques. The LMR was calculated by dividing the lymphocyte count by the monocyte count. The platelet-to-lymphocyte ratio (PLR) was computed by dividing the platelet count by the lymphocyte count. The neutrophil-to-lymphocyte ratio (NLR) was obtained by dividing the neutrophil count by the lymphocyte count. The systemic inflammatory index (SII) was calculated using the formula (neutrophil × platelet)/lymphocyte. Transthoracic echocardiography was conducted using ultrasound equipment based on the guidelines of the American Society of Echocardiography [21]. A LVEF of at least 45% was defined as preserved ejection fraction [22].

### 2.3 Outcomes

The primary endpoint was designated as overall mortality. The secondary endpoint focused on cardiovascular mortality. Data on outcomes were collected from the death registration system and through follow-up telephone calls. The duration of follow-up was calculated as the time between the admission date and either the date of death or the last follow-up. The follow-up period concluded in December 2023.

### 2.4 Statistical Analysis

Baseline characteristics are presented as proportions and frequencies for the categorical variables, while continuous variables are presented as the median with interquartile ranges (IQR) for non-normally distributed data or as the mean ± standard deviation (SD) for normally distributed data. Categorical variables are reported as counts and percentages. Participants were divided into tertiles based on the calculated LMR. To compare differences among groups for continuous variables, one-way analysis of variance (ANOVA) or the Kruskal–Wallis test was employed. For categorical variables, the Pearson chi-square test was used as appropriate. NT-proBNP and the SII were analyzed after logarithmic transformation [log (NT-proBNP), log (SII)]. Linear regression was conducted to assess the relationships between age and the LMR, NLR, PLR, and log (SII) using Pearson’s correlation test.

The Kaplan-Meier method was implemented to illustrate the survival rates excluding all-cause mortality and cardiovascular mortality. The relationships between the LMR and all-cause mortality or cardiovascular death were depicted using spline curves. We utilized three multivariable Cox regression models to adjust for potential confounders affecting outcomes: the first model was unadjusted; the second model adjusted for age (continuous), gender (male/female), and BMI (continuous); the third model further adjusted for CVD history (yes/no), AF history (yes/no), systolic blood pressure (SBP, continuous), HbA1c (continuous), serum creatinine (continuous), LDL-c (continuous), log(NT-proBNP) (continuous), and smoking status (current/former or never).

Mediation analyses were performed using the Stata package “medeff” and the R package “mediation” to evaluate the mediating roles of the LMR, NLR, PLR, and log(SII) in the relationship between age and the prognosis of HFpEF. Sensitivity analysis for mediation was conducted using the “medsens” package in Stata. Partial mediation was defined as a situation where both the total and indirect effects were statistically significant. Statistical analyses were performed using SPSS 26.0 (IBM, Armonk, NY, USA), Stata 16.0 (Stata Corp LLC, College Station, TX, USA), and R version 4.2.1 (R Foundation for Statistical Computing, Vienna, Austria). A two-sided *p-*value of less than 0.05 was considered statistically significant.

## 3. Result

### 3.1 Baseline Features

The final sample comprised 1274 inpatients diagnosed with HFpEF (**Supplementary Fig. 1**). Among these patients, 66.1% were male, with a mean age of 66.20 ± 10.72 years and an average BMI of 24.26 ± 3.41 kg/m^2^ (Table 1). The participants were divided into tertiles based on their baseline LMR: tertile 1 (<2.62), tertile 2 (2.62–3.94), and tertile 3 (≥3.94). As demonstrated in Table 1, patients in the first tertile had an advanced age, a higher proportion of males, and exhibited elevated levels of BNP or NT-proBNP (all *p* < 0.001). With a median follow-up period of 4.9 years, those in tertile one experienced the highest rate of all-cause mortality at 20.8%. In contrast, the mortality rates for tertiles two and three were 11.1% and 7.3%, respectively (*p* < 0.001). A similar trend was observed for cardiovascular mortality, with rates of 11.7% in the first tertile, 5.1% in the second tertile, and 2.6% in the third tertile (*p* < 0.001).

**Table 1.** **Baseline characteristics of HFpEF**.

Variables	HFpEF	Tertile 1	Tertile 2	Tertile 3	*p*-value
	(n = 1274)	(n = 418)	(n = 434)	(n = 422)	
Age, years	66.20 (10.72)	67.79 (11.20)	66.68 (10.74)	64.14 (9.86)	<0.001
Male, % (n)	66.1% (842)	75.1% (314)	68.0% (295)	55.20% (233)	<0.001
BMI, kg/m^2^	24.26 (3.41)	23.66 (3.40)	24.68 (3.51)	24.72 (3.23)	<0.001
SBP, mmHg	133.68 (20.59)	135.24 (21.66)	134.38 (20.56)	131.41 (19.34)	0.018
CVD history, % (n)	85.6% (1091)	83.0% (347)	85.7% (372)	88.2% (372)	0.105
AF history, % (n)	7.5% (95)	11.5% (48)	6.5% (28)	4.5% (19)	<0.001
Smoking, % (n)	40.2% (512)	42.3% (177)	44.7% (194)	33.4% (141)	0.002
LDL, mmol/L	2.67 (0.87)	2.56 (0.84)	2.66 (0.83)	2.80 (0.84)	0.001
HbA1c, %	6.00 (1.20)	6.38 (1.28)	6.43 (1.31)	6.40 (1.29)	0.869
Serum creatinine, µmol/L	82 (68, 102)	91 (74, 120)	81 (68, 99)	77 (65, 90)	<0.001
Log (NT-proBNP)	1.67 (0.79, 1.81)	1.70 (0.71, 1.80)	1.67 (0.79, 1.81)	0.96 (0.78, 1.80)	0.180
Death, % (n)	13.0% (166)	20.8% (87)	11.1% (48)	7.3% (31)	<0.001
Cardiovascular death	6.4% (82)	11.7% (49)	5.1% (22)	2.6% (11)	<0.001

Continuous variables such as age, BMI, SBP, LDL-c, and HbA1c are expressed as the mean (SD). In contrast, continuous variables, such as creatinine and log(NT-proBNP) are shown as the median (interquartile range, IQR). Categorical variables are reported as counts and percentages. HFpEF, heart failure with preserved ejection fraction; BMI, body mass index; SBP, systolic blood pressure; CVD, cardiovascular disease; AF, atrial fibrillation; LDL-c, low-density lipoprotein cholesterol; NT-proBNP, N terminal pro-B-type natriuretic peptide; NLR, neutrophil to lymphocyte ratio; PLR, platelet to lymphocyte ratio; LMR, lymphocyte to monocyte ratio; SII, systemic inflammatory index; SD, standard deviation; IQR, interquartile range.

### 3.2 Relationship Between Age and Prognosis of HFpEF

With a median follow-up duration of 4.9 years, there were 166 recorded deaths, of which 82 were due to cardiovascular causes (Table 1). The spline curves illustrated a clear trend, indicating that older patients faced a higher risk of both all-cause and cardiovascular mortality (**Supplementary Fig. 2**). Three Cox regression models were employed to quantitatively analyze the association between age and the prognosis of HFpEF. In the third model, each one-unit increase in SD for age was associated with a 1.98-fold increase in the risk of overall mortality (95% CI (1.66, 2.35); *p* < 0.001) and a 1.73-fold increase in the risk of mortality due to cardiovascular disease (95% CI (1.36, 2.21); *p* < 0.001) (**Supplementary Table 1**). Consequently, a significant correlation was established between age and the prognosis of HFpEF, suggesting that advancing age is linked to an elevated risk of adverse clinical outcomes in patients with this condition.

### 3.3 Relation Between LMR and Prognosis of HFpEF

**Fig. 1.**
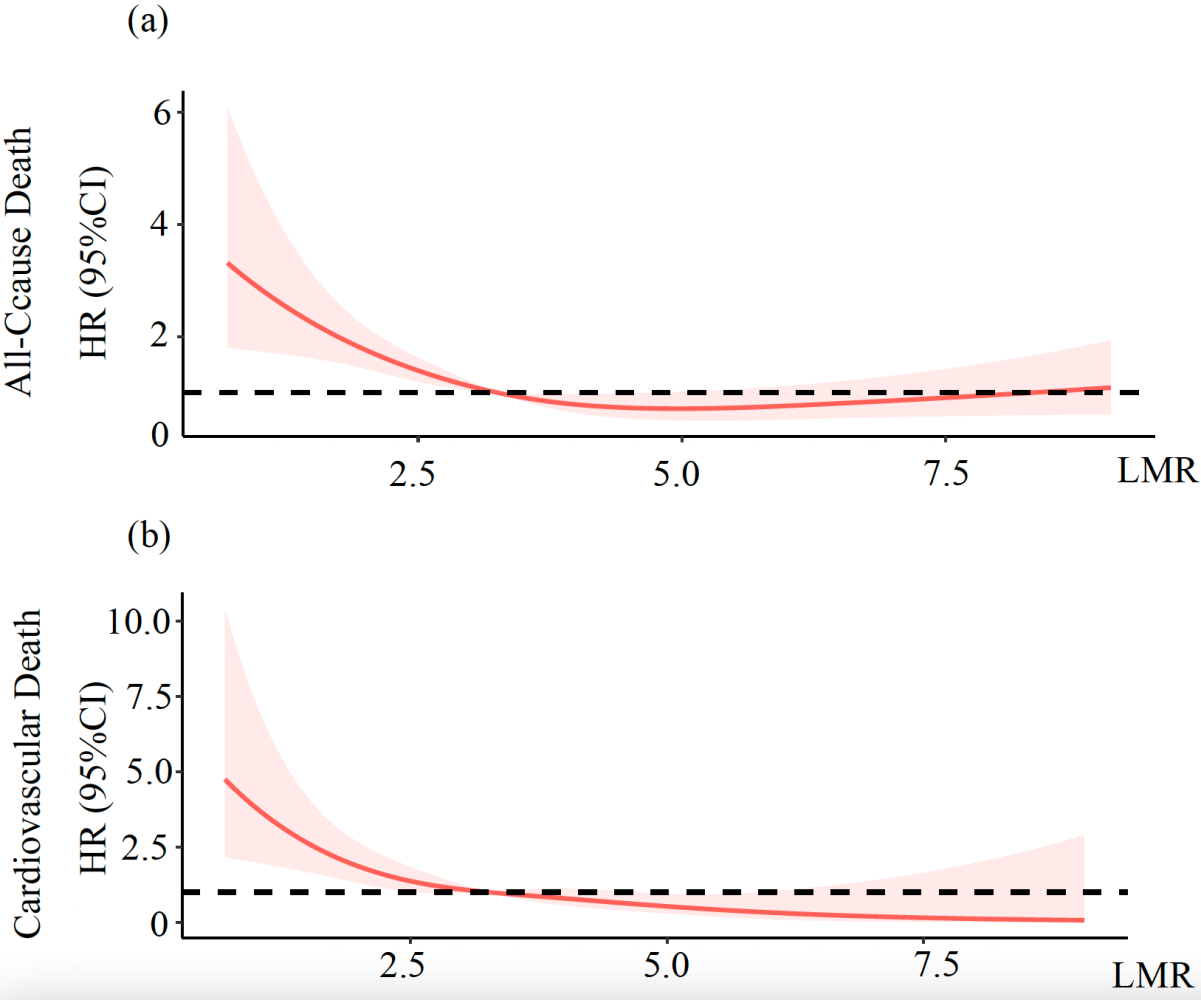
**Risk of all-cause death (a) and cardiovascular death (b) across the full range of the LMR**. The fully adjusted HRs for all-cause mortality (a) and cardiovascular mortality (b) based on the LMR are displayed. Each HR was calculated using the median LMR value of 3.26 as the reference point. The adjustments for these HRs considered several factors, including age, gender, BMI, SBP, LDL-c, HbA1c, serum creatinine, log(NT-proBNP), history of AF, and history of CVD. The red solid line depicts the HR of the LMR across its full range. The pink shaded area symbolizes the 95% CI for the HRs. The black dotted line represents the reference line at HR = 1. LMR, lymphocyte to monocyte ratio; HR, hazard ratio; CI, confidence interval.

The restricted cubic spline analysis depicted in Fig. 1 revealed that lower LMR values were associated with higher odds of both all-cause mortality and cardiovascular mortality. Thus, multivariate Cox regression analyses were performed to investigate the association between the LMR and the prognosis of HFpEF further. As illustrated in Table 2, patients in the second tertile had a 45% lower risk of overall mortality compared to those in the first tertile (95% CI (0.38–0.80); *p* < 0.001). Similarly, patients in the third tertile demonstrated a 58% reduction in risk compared to those in the first tertile (95% CI (0.27–0.65); *p* = 0.002).

**Table 2.** **Risk of overall death and cardiovascular death in inpatients with HFpEF categorized by the LMR**.

Group		Events/n	Model 1		Model 2		Model 3	
			HR (95% CI)	*p*-value	HR (95% CI)	*p*-value	HR (95% CI)	*p*-value
Overall death								
	LMR <2.62	87/418	Reference		Reference		Reference	
	2.62 ≤ LMR < 3.94	48/434	0.47 (0.33, 0.67)	<0.001	0.50 (0.35, 0.72)	<0.001	0.55 (0.38, 0.80)	<0.001
	LMR ≥3.94	31/422	0.28 (0.19, 0.43)	<0.001	0.36 (0.24, 0.56)	<0.001	0.42 (0.27, 0.65)	0.002
	*p* for trend		<0.001		<0.001		<0.001	
Cardiovascular death								
	LMR <2.62	49/418	Reference		Reference		Reference	
	2.62 ≤ LMR < 3.94	22/434	0.39 (0.23, 0.64)	<0.001	0.39 (0.24, 0.66)	<0.001	0.42 (0.25, 0.71)	<0.001
	LMR ≥3.94	11/422	0.18 (0.09, 0.35)	<0.001	0.21 (0.11, 0.41)	<0.001	0.23 (0.12, 0.46)	<0.001
	*p* for trend		<0.001		<0.001		<0.001	

Cox proportional hazard models were fully adjusted by age, gender, BMI, SBP, LDL-c, HbA1c, serum creatinine, log(NT-proBNP), CVD history, and AF history at baseline.

A comparable trend was observed for cardiovascular mortality. Patients in tertile two had a 58% lower risk (95% CI (0.25–0.71); *p* < 0.001) compared to those in tertile one, while those in tertile three had a 77% lower risk (95% CI (0.12–0.46); *p* < 0.001). Specifically, as the LMR decreased, the likelihood of adverse clinical outcomes increased. Additionally, the relationships between other systemic inflammatory markers, namely NLR, PLR, and logSII, and clinical outcomes were also examined. Patients were categorized into tertiles for these markers and analyzed using the Kaplan-Meier method (**Supplementary Fig. 3**). In the multivariate Cox regression analyses presented in **Supplementary Table 2**, significant associations were found between the NLR, PLR, and logSII with both all-cause mortality and cardiovascular mortality (all *p* for trend < 0.05).

### 3.4 Mediation Analysis

Based on prior research and known underlying mechanisms, we hypothesized that the LMR could vary with age and be associated with mortality for patients with HFpEF. To evaluate the mediating role of the LMR in the relationship between age and mortality for HFpEF, we initially performed a linear correlation analysis using Pearson’s correlation test. This analysis revealed a negative correlation, indicating that the LMR decreased as the age increased (β = –0.021, *p* < 0.001; **Supplementary Fig. 4**).

**Fig. 2.**
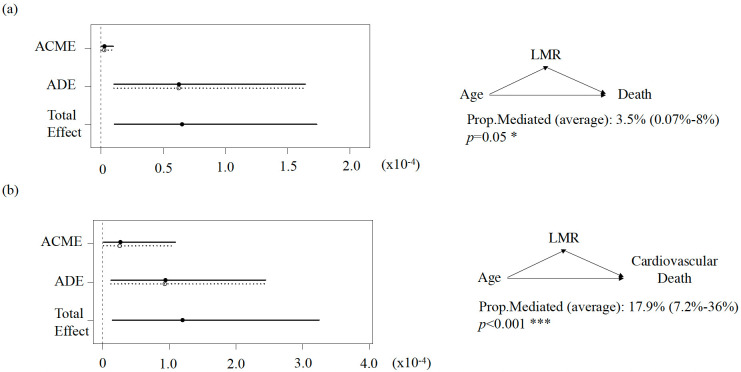
**Median analysis to evaluate the mediating role of the LMR in the association between age and all-cause death (a) and cardiovascular death (b) for HFpEF**. ACME, average causal mediation effect (indirect effect); ADE, average mediation effect (direct effect); Prop. Mediated, the proportion of the mediating effect.

Subsequently, mediation analysis revealed that the LMR contributed to a mediation effect of 3.5% (95% CI (0.07%–8%); *p* = 0.05; **Supplementary Table 3**) in the relationship between age and all-cause mortality. For cardiovascular mortality, the LMR accounted for a mediation proportion of 17.9% (95% CI (7.2%–36%); *p* < 0.001; Fig. 2). Sensitivity analysis was performed to check the robustness of the mediation analysis (**Supplementary Table 4, Supplementary Fig. 5**). Results of the mediation analyses between the rest of systemic inflammatory markers and clinical outcomes were displayed in **Supplementary Fig. 6**

## 4. Discussion

In this longitudinal study, the LMR was found to have an exacerbating effect on the outcomes of HFpEF. Specifically, the LMR mediated 17.9% of the association between age and cardiovascular mortality in patients with HFpEF. These findings suggest that the LMR could serve as an economically accessible biomarker for predicting the prognosis of HFpEF. Additionally, the results provide insights into the mechanisms underlying longevity, particularly through the lenses of immunosenescence and inflamm-aging.

HFpEF is predominantly a condition affecting older adults, with most patients being over 65 years of age. Data from the MAGGIC meta-analysis and the Candesartan in Heart failure-Assessment of moRtality and Morbidity (CHARM) program indicate that older patients with heart failure experience higher mortality rates compared to younger individuals [23, 24]. Specifically, patients aged 85 and older have a 5.9-fold increased risk of mortality compared to those aged 55 and younger [4, 23, 24]. The inflammation associated with heart failure is believed to be linked to several factors, including heightened oxidative stress, reduced autophagy and mitophagy, increased DNA damage, mitochondrial dysfunction, and cellular senescence. These processes contribute to cell death, which activates the innate immune system and provokes the production of inflammatory cytokines, thereby exacerbating the effects of cell death. Over the past decade, the Canakinumab Anti-Inflammatory Thrombosis Outcomes Study (CANTOS) trial has demonstrated that anti-inflammatory treatment with canakinumab is associated with a reduced risk of heart failure-related mortality in patients with elevated high-sensitivity C-reactive protein (hs-CRP) levels [25]. More recently, research into the mechanisms behind the benefits of sodium-glucose cotransporter 2 (SGLT2) inhibitors in HFpEF patients has shown that these medications can reduce epicardial adipose tissue and modify adipokine signaling, potentially leading to decreased inflammation and oxidative stress [26, 27]. Additionally, the STEP-HFpEF trial found that semaglutide significantly improved symptoms and reduced inflammation in participants with HFpEF who also had obesity [28]. Given these insights into the role of inflammation in heart failure, our study introduced the LMR as a systemic inflammatory marker that is readily accessible through routine clinical blood tests, offering a valuable tool for predicting the prognosis of HFpEF.

Although the exact mechanisms linking the LMR to survival in HFpEF are not completely understood, several explanations can be proposed. Firstly, lymphocytes play a crucial role in activating the host immune response and maintaining immune surveillance. Lymphocytopenia, or a low count of lymphocytes, may lead to inappropriate immune responses as individuals age. A deficiency in peripheral lymphocytes can result in the accumulation of senescent cells and unresolved inflammation [29]. Secondly, elevated circulating monocyte levels indicate increased peripheral inflammation. These monocytes can further differentiate into macrophages, which are likely linked to the development of HFpEF and its precursor, asymptomatic left ventricular diastolic dysfunction [19]. Our data show that the LMR changes with age, supporting our hypothesis that the LMR is independently associated with the prognosis of HFpEF. This finding aligns with the notion that inflamm-aging may play a significant role in the progression of HFpEF [30, 31, 32].

In addition to the LMR, other systemic inflammatory markers, such as the NLR, PLR, and SII, are also significantly associated with the prognosis of HFpEF. This highlights the significance of systemic inflammation in the progression of HFpEF. Research by Tamaki *et al*. [33] indicated that in patients with HFpEF who were hospitalized for acute decompensated heart failure, the combination of the NLR and PLR proved to be effective in predicting cardiac mortality after discharge. Similarly, Curran *et al*. [34] found that elevated NLR levels were significantly correlated with NT-proBNP levels, as well as poorer outcomes in heart failure patients. An increase in the NLR may be particularly useful for identifying high-risk patients with heart failure [34]. Furthermore, Liu *et al*. [30] reported that both the LMR and PLR served as independent prognostic factors for patients with chronic heart failure (CHF). In our study, multivariate Cox regression analyses and restricted cubic spline analyses demonstrated that the NLR, PLR, and SII, in addition to the LMR, possess strong predictive value for the prognosis of HFpEF. In a cross-sectional study, the LMR was reported to have a good diagnostic value for the incidence of HFpEF [35]. Silva *et al*. [31] suggested that low LMR values are independently related to a higher risk of 6-month death after an episode of acute heart failure. These findings collectively highlight that systemic inflammation plays a crucial role in the progression of HFpEF, underscoring the importance of maintaining a proper balance between proinflammatory and anti-inflammatory factors in patients with this condition.

The comorbidity–inflammation model characterizes HFpEF as a result of a systemic proinflammatory state triggered by various comorbidities. This inflammatory condition leads to endothelial dysfunction, coronary microvascular dysfunction, and alterations in cardiac structure and function, ultimately resulting in HFpEF [36]. Older adults are particularly susceptible to a combination of multiple morbidities that contribute to frailty; thus, understanding the relationship between heart disease and frailty is essential [37]. Skeletal muscle may play a critical role in the interplay between aging, sarcopenia, inflammation, and cardiovascular diseases [37, 38]. Indeed, research by Adams *et al*. [39] highlighted that in HFpEF patients, skeletal muscle is characterized by increased proteolysis associated with systemic inflammation and diminished exercise capacity, as well as disturbances in energy metabolism. Moreover, systemic inflammation and coronary microvascular endothelial dysfunction are of great importance in regulating the impacts of extracardiac comorbidities, such as metabolic disorders, hypertension, and renal impairment, on left ventricular remodeling and functional decline [39]. In this context, we investigated the mechanistic effects of immunosenescence and inflamm-aging on HFpEF, positing that aging fundamentally alters inflammatory pathways both in the heart and systemically, thereby contributing to the development of this condition [40].

Our study presents several important clinical implications. First, we found that systemic inflammatory markers, such as the LMR, NLR, PLR, and SII, are associated with the prognosis of HFpEF. These markers may serve as valuable prognostic indicators for HFpEF. Secondly, our study had a median follow-up period of 4.9 years, demonstrating that the LMR possesses long-term prognostic significance for patients with HFpEF. Thirdly, our mediation analysis shed light on the critical role of inflammation in driving poor prognosis among older patients with HFpEF from a clinical perspective, implicating that immunosenescence and inflamm-aging could be significant mechanisms underlying the development of HFpEF.

### Limitations

However, several limitations of the study should be noted. Firstly, as an observational cohort study, this analysis cannot establish causal relationships and may be influenced by residual confounding factors. Secondly, patients were grouped into tertiles based on the LMR, which resulted in unequal baseline demographics among the groups. To address this, we employed three Cox regression models to adjust for potential confounders. Thirdly, this research was conducted in a single-center cohort in Southeast China, which limits generalizability. Fourthly, only a single baseline measurement of the LMR was included; future studies should incorporate dynamic changes over time. Fifthly, external validation was necessary; however, due to the limited data available from other partner hospitals, we were unable to incorporate this validation into the study. Sixthly, the blood routine was missed for 384 out of 2448 HFpEF patients, which inevitably introduced selection bias, although our baseline was generally balanced (not shown). Further validations are needed for a more solid conclusion.

## 5. Conclusions

In summary, our findings suggest that systemic inflammatory markers, particularly the LMR, NLR, PLR, and SII, are strongly associated with mortality outcomes for HFpEF. Notably, the LMR appears to act as a mediator for age-related cardiovascular mortality. We propose that a biomarker signature derived from routine blood tests could be valuable in monitoring the progression of systemic inflammation during aging and the advancement of HFpEF.

## Availability of Data and Materials

The data of this study are not publicly available yet as the research is still ongoing. If needed, please contact zhuangxd3@mail.sysu.edu.cn (Xiaodong Zhuang) via email.

## Supplementary Material

Supplementary material associated with this article can be found, in the online version, at https://doi.org/10.31083/RCM45403.
