# Supplementary material for: Lymphocyte-To-Monocyte Ratio is Partially Mediated in Age-Related Cardiovascular Mortality in HFpEF: Immunosenescence, Inflamm-Aging, and Longevity
Source: Rev Cardiovasc Med. 2026 Feb 11;27(2):45403. doi: 10.31083/RCM45403 (PMC12959978; doi:10.31083/RCM45403)
Supplement: Supplementary file 1 [file 2153-8174-27-2-45403-s1.zip › Supplementary Material.docx]

Supplementary Fig. 1: inclusion and exclusion criteria of this study.

RED-CARPET trial HF patients with outcomes and at least one UCG (N=2448)

- LVEF missed or less than 45% (N=631)
- blood routine missed (N=384)
- NT-proBNP or BNP missed(N=66)
- smoking history missed (N=48)
- severe valve disease (N=45)

exclusion

HFpEF patients included

(N=1274)

RED-CARPET trial: the Real-world Data of Cardiometabolic ProtEcTion study; HF: heart failure; UCG: ultrasound cardiogram; LVEF: left ventricular ejection fraction; NT-proBNP: N terminal pro B-type natriuretic peptide; BNP: B-type natriuretic peptide; HFpEF: heart failure with preserved ejection fraction.

 Supplementary Fig. 2: risk of all-cause death (a) and cardiovascular death (b) according to age.

Fully adjusted hazard ratios (HRs) of all-cause death (a) and cardiovascular death (b) by age. Each HR was computed with the median age (66.50 years old) as the reference. The HRs adjusted by gender, BMI, SBP, LDL-c, HbA1c, serum creatine, log(NT-proBNP), AF history, and CVD history. The red solid line represents the HR of age across the whole range. The pink area represents the 95% confidence interval (CI). The black dotted line is the reference line as HR=1.

Supplementary Fig. 3: Kaplan-Meier curve of LMR, NLR, PLR, and logSII according to corresponding tertiles.

1. death-free survival rate according to tertiles of LMR; (b) cardiovascular death-free survival rate according to tertiles of LMR; (c) death-free survival rate categorized by tertiles of NLR; (d) cardiovascular death-free survival rate according to tertiles of NLR; (e) death-free survival rate based on tertiles of PLR; (d) cardiovascular death-free survival rate based on tertiles of PLR; (g) death-free survival rate identified by tertiles of logSII; (h) cardiovascular death-free survival rate identified by tertiles of logSII. T1: tertile 1; T2: tertile 2; T3: tertile 3. LMR:lymphocyte to monocyte ratio; NLR: neutrophil to lymphocyte ratio; PLR: platelet to lymphocyte ratio; SII: systemic inflammatory index.

Supplementary Fig. 4: scatter-plots between LMR and age

On Pearson’s correlation test, there was a significant correlation (p=0.00053) between age and LMR. LMR: lymphocyte to monocyte ratio; β: beta coefficient.

Supplementary Fig. 5: graphical presentation of the results from the sensitivity analysis of LMR’s role in the association between age and cardiovascular death.

ACME: average causal mediation effect (indirect effect).

Supplementary Fig. 6: the mediating role of NLR, PLR, or logSII in the association of age with clinical outcomes of HFpEF.

Mediation role of NLR in the association of age with all-cause death (a), and age with cardiovascular death (b); Mediation role of PLR in the association of age with all-cause death (c), and age with cardiovascular death (d); and Mediation role of logSII in the association of age with all-cause death (e), and cardiovascular death (f). Causal mediation effect was fully adjusted by age, gender, body mass index, systolic blood pressure, low-density lipoprotein, glycosylated hemoglobin, serum creatine, log(NT-proBNP), CVD history, AF history at baseline. NLR neutrophil to lymphocyte ratio; PLR: platelet to lymphocyte ratio; SII: systemic inflammatory index; Prop.Mediated: the proportion of the mediating effect.

Supplementary Table 1: Correlation of age and all-cause death (a) and cardiovascular death (b).

(a)

|  | Model 1 | | Model 2 | | Model 3 | |
| --- | --- | --- | --- | --- | --- | --- |
|  | HR | *p* | HR | *p* | HR | *p* |
| age/SD | 1.95 (1.65, 2.31) | <0.001 | 2.01 (1.69, 2.38) | <0.001 | 1.98 (1.66, 2.35) | <0.001 |

(b)

|  | Model 1 | | Model 2 | | Model 3 | |
| --- | --- | --- | --- | --- | --- | --- |
|  | HR | *p* | HR | *p* | HR | *p* |
| age/SD | 1.76 (1.39, 2.22) | <0.001 | 1.79 (1.41, 2.27) | <0.001 | 1.73 (1.36, 2.21) | <0.001 |

Risk of all-cause death (a) and cardiovascular death (b) according to age/SD. Model 1 was unadjusted. Model 2 was adjusted for age, gender, and BMI. Model 3 was fully adjusted by age, gender, body mass index, systolic blood pressure, low-density lipoprotein, glycosylated hemoglobin, serum creatine, log(NT-proBNP), CVD history, AF history at baseline based on model 2. SD: standard deviation; HR: hazard ratio.

Supplementary Table 2: Risk of all-cause death and cardiovascular death in inpatients with HFpEF according to NLR, PLR, logSII.

(a)

| Groups | Events/N | Model 1 | | Model 2 | | Model 3 | |
| --- | --- | --- | --- | --- | --- | --- | --- |
|  |  | HR | *p* | HR | *p* | HR | *p* |
| NLR<1.91 | 33/415 | reference | | reference | | reference | |
| 1.91=<NLR<2.99 | 52/439 | 1.48 (0.96,2.29) | 0.078 | 1.17 (0.75, 1.83) | 0.476 | 1.02 (0.65, 1.61) | 0.916 |
| NLR>=2.99 | 81/420 | 2.70 (1.80,4.05) | <0.001 | 2.21 (1.41, 3.23) | <0.001 | 1.85 (1.20, 2.83) | 0.005 |
| P for trend | - | <0.001 | | <0.001 | | <0.001 | |

(b)

| Groups | Events/N | Model 1 | | Mode 2 | | Model 3 | |
| --- | --- | --- | --- | --- | --- | --- | --- |
|  |  | HR (95%CI) | *p* | HR (95%CI) | *p* | HR (95%CI) | *p* |
| NLR<1.91 | 16/415 | reference | | reference | | reference | |
| 1.91=<NLR<2.99 | 20/439 | 1.17 (0.61,2.26) | 0.634 | 0.98 (0.50, 1.90) | 0.953 | 0.87 (0.44, 1.70) | 0.678 |
| NLR>=2.99 | 46/420 | 3.13 (1.77,5.53) | <0.001 | 2.63 (1.47, 4.71) | 0.001 | 2.37 (1.30, 4.31) | 0.005 |
| P for trend | -- | <0.001 | | <0.001 | | <0.001 | |

(c)

| Groups | Events/N | Model 1 | | Model 2 | | Model 3 | |
| --- | --- | --- | --- | --- | --- | --- | --- |
|  |  | HR (95%CI) | *p* | HR (95%CI) | *p* | HR (95%CI) | *p* |
| PLR<106.73 | 49/420 | reference | | reference | | reference | |
| 106.73=<PLR<151.49 | 44/434 | 0.87 (0.58,1.30) | 0.494 | 1.17 (0.75, 1.83) | 0.476 | 0.74 (0.49, 1.13) | 0.164 |
| PLR>=151.49 | 73/420 | 1.57 (1.01,2.25) | 0.015 | 1.43 (0.99, 2.06) | 0.057 | 1.26 (0.87, 1.84) | 0.219 |
| P for trend | - | 0.003 | | <0.009 | | 0.025 | |

(d)

| Groups | Events/N | Mode 1 | | Mode 2 | | Mode 3 | |
| --- | --- | --- | --- | --- | --- | --- | --- |
|  |  | HR (95%CI) | *p* | HR (95%CI) | *p* | HR (95%CI) | *p* |
| PLR<106.73 | 23/420 | reference | | reference | | reference | |
| 106.73=<PLR<151.49 | 20/434 | 0.85 (0.46,1.54) | 0.584 | 0.80 (0.44, 1.46) | 0.473 | 0.75 (0.41, 1.38) | 0.358 |
| PLR>=151.49 | 39/420 | 1.78 (1.06,2.99) | 0.028 | 1.66 (0.99, 2.79) | 0.056 | 1.54 (0.91, 2.61) | 0.110 |
| P for trend | - | 0.011 | | 0.018 | | 0.029 | |

(e)

| Groups | Events/N | Model 1 | | Model 2 | | Model 3 | |
| --- | --- | --- | --- | --- | --- | --- | --- |
|  |  | HR (95%CI) | *p* | HR (95%CI) | *p* | HR (95%CI) | *p* |
| logSII<2.63 | 49/424 | reference | | reference | | reference | |
| 2.63=<logSII<2.85 | 45/431 | 0.91 (0.61,1.37) | 0.663 | 0.82 (0.55, 1.23) | 0.334 | 0.78 (0.52, 1.18) | 0.244 |
| logSII>=2.85 | 72/419 | 1.63 (1.14,2.35) | 0.008 | 1.56 (1.08, 2.25) | 0.017 | 1.38 (0.95, 2.00) | 0.087 |
| P for trend | - | 0.003 | | 0.002 | | 0.013 | |

(f)

| Groups | Events/N | Model 1 | | Model 2 | | Model 3 | |
| --- | --- | --- | --- | --- | --- | --- | --- |
|  |  | HR (95%CI) | *p* | HR (95%CI) | *p* | HR (95%CI) | *p* |
| logSII<2.63 | 26/424 | reference | | reference | | reference | |
| 2.63=<logSII<2.85 | 17/431 | 0.65 (0.35,1.20) | 0.165 | 0.59 (0.32, 1.09) | 0.090 | 0.55 (0.30, 1.01) | 0.055 |
| logSII>=2.85 | 39/419 | 1.65 (1.00,2.70) | 0.049 | 1.59 (0.97, 2.62) | 0.068 | 1.44 (0.87, 2.39) | 0.156 |
| P for trend | - | 0.004 | | 0.002 | | 0.005 | |

Risk of all-cause death (a) and cardiovascular death (b) identified by NLR, all-cause death (c) and cardiovascular death (d) identified by PLR, all-cause death (e) and cardiovascular death (f) identified by logSII. Model 1 was unadjusted. Model 2 was adjusted for age, gender, and BMI. Model3 was fully adjusted by age, gender, body mass index, systolic blood pressure, low-density lipoprotein, glycosylated hemoglobin, serum creatine, log(NT-proBNP), CVD history, AF history at baseline. HR: hazard ratio; CI: confidence interval; NLR: neutrophil to lymphocyte ratio; PLR: platelet to lymphocyte ratio; SII: systemic inflammatory index.

Supplementary Table 3: estimates of the mediation analysis of LMR in the association between age and clinical outcomes of HFpEF.

| Outcome | Mediator | Total effect (x10^-4^) | ADE (x10^-4^) | ACME(x10^-4^) | Mediated (95%CI), % | *p* |
| --- | --- | --- | --- | --- | --- | --- |
| All-cause death | LMR | 1.70 (0.16, 6.7) | 1.62 (0.15, 6.5) | 0.086 (0.007, 0.4) | 3.5 (0.07-8) | 0.05 |
| Cardiovascular death | LMR | 2.88 (0.14, 14) | 2.38 (0.097, 13) | 0.51 (0.02, 2.8) | 17.9 (7.2-36) | <0.001 |

ACME: average causal mediation effect (indirect effect); ADE: average mediation effect (direct effect); LMR: lymphocyte to monocyte ratio.

Supplementary Table 4: results of the sensitivity analysis of LMR in the association between age and clinical outcomes of HFpEF.

|  | Outcomes | |
| --- | --- | --- |
| Sensitivity results | All-cause death | Cardiovascular death |
| Rho at which ACME=0 | -0.2 | -0.4 |
| R^2_M*R^2_Y* at which ACME=0: | -0.04 | -0.16 |
| R^2_M*R^2_Y~ at which ACME=0: | -0.016 | -0.064 |

ACME: average causal mediation effect (indirect effect).
